# Sex differences in the genetic regulation of the blood transcriptome response to glucocorticoid receptor activation

**DOI:** 10.1038/s41398-021-01756-2

**Published:** 2021-12-13

**Authors:** Sarah R. Moore, Thorhildur Halldorsdottir, Jade Martins, Susanne Lucae, Bertram Müller-Myhsok, Nikola S. Müller, Charlotte Piechaczek, Lisa Feldmann, Franz Joseph Freisleder, Ellen Greimel, Gerd Schulte-Körne, Elisabeth B. Binder, Janine Arloth

**Affiliations:** 1 BC Children’s Hospital Research Institute and Centre for Molecular Medicine and Therapeutics, Vancouver, BC Canada; 2 Reykjavik University, Reykjavik, Iceland; 3 Department of Translational Research in Psychiatry, Max Planck Institute of Psychiatry, Munich, 80804 Germany; 4 Institute of Computational Biology, Helmholtz Zentrum München, Neuherberg, 85764 Germany; 5 Department of Child and Adolescent Psychiatry, Psychosomatics and Psychotherapy, Ludwig-Maximilians-University (LMU) Hospital, Munich, Germany; 6 KBO Heckscher-Klinikum, Munich, Germany; 7 Department of Psychiatry and Behavioral Sciences, Emory University School of Medicine, Atlanta, GA 30322 USA

**Keywords:** Genomics, Predictive markers

## Abstract

Substantial sex differences have been reported in the physiological response to stress at multiple levels, including the release of the stress hormone, cortisol. Here, we explore the genomic variants in 93 females and 196 males regulating the initial transcriptional response to cortisol via glucocorticoid receptor (GR) activation. Gene expression levels in peripheral blood were obtained before and after GR-stimulation with the selective GR agonist dexamethasone to identify differential expression following GR-activation. Sex stratified analyses revealed that while the transcripts responsive to GR-stimulation were mostly overlapping between males and females, the quantitative trait loci (eQTLs) regulation differential transcription to GR-stimulation was distinct. Sex-stratified eQTL SNPs (eSNPs) were located in different functional genomic elements and sex-stratified transcripts were enriched within postmortem brain transcriptional profiles associated with Major Depressive Disorder (MDD) specifically in males and females in the cingulate cortex. Female eSNPs were enriched among SNPs linked to MDD in genome-wide association studies. Finally, transcriptional sensitive genetic profile scores derived from sex-stratified eSNPS regulating differential transcription to GR-stimulation were predictive of depression status and depressive symptoms in a sex-concordant manner in a child and adolescent cohort (*n* = 584). These results suggest the potential of eQTLs regulating differential transcription to GR-stimulation as biomarkers of sex-specific biological risk for stress-related psychiatric disorders.

## Introduction

Robust sex differences have been reported for stress-related psychiatric disorders, including mood and anxiety disorders, schizophrenia, and post-traumatic stress disorder (PTSD) [1–4]. Beyond prevalence rates, consistent sex differences are observed in the age of onset, symptomology, comorbidities, and responses to medication [1, 3–5]. For instance, major depressive disorder (MDD) demonstrates higher prevalence rates in women than in men [3] and women exhibit heightened vulnerability to mood symptoms in association with stress-induced inflammatory processes [6]. Despite the accumulating evidence for sex differences in stress-related pathogenesis of psychiatric conditions, the etiological mechanisms responsible for these differences are not well understood. Elucidating sex-related factors that moderate stress susceptibility is critical for targeted prevention and treatment strategies.

Evidence suggests that a dysregulation of the hypothalamic-pituitary-adrenal (HPA) axis contributes to vulnerability to stress [6–9]. Exposure to stressful environments or threat leads to the activation of the HPA axis, with the release of hypothalamic corticotropin-releasing hormone (CRH) that in turn stimulates the release of adrenocorticotropin from the pituitary into the peripheral circulation. This leads to the release of glucocorticoids (GC) from the adrenal cortex. GCs bind to mineralo- and glucocorticoid receptors (GR), with the GR regulating biological adaptations to chronic stressors [10–12]. The GR is highly expressed in most tissues both peripherally and centrally. Activation of GR by GCs causes the translocation of GR from the cytoplasm to the nucleus [13]. There it binds to glucocorticoid response elements (GREs) and regulates gene expression. The resulting biological cascade has broad biological effects, initiating physiological changes in the body for adaptation to threat, and also providing negative feedback regulation to the brain for recovery [14].

Sex differences in the stress response have been amply demonstrated at the physiological, hormonal, and neuroinflammatory levels [6, 8]. In human studies, sex differences have been reported in both physiological and emotional responses to standardized stress tests, such as the Trier Social Stress Test [15–17]. Importantly, these stress response indices demonstrate abnormalities following exposure to childhood trauma [18] and in stress-related psychiatric disorders [19]. Thus, a better understanding of sex differences in the stress response may inform the sex-biased pathways to stress- and trauma-related psychiatric disorders.

Sex differences in the stress response have largely been attributed to gonadal hormone changes. Sex chromosomes determine gonad development and gonadal hormones then alter regulatory pathways affecting the transcriptome and epigenome in sex-specific ways [20]. Indeed, the transcriptome [21–23] and epigenome [24, 25] are highly sex-specific. Animal models have shown that transcriptional changes due to stress exposure are sex-specific in the hippocampus [26] and hypothalamus [27]. Sex-specificity of the transcriptome extends to transcriptional signatures of MDD in humans [28]. For instance, MDD-associated transcriptional networks across brain regions are highly disparate between males and females, with sex-stratified results converging with sex differences in a mouse model of chronic social stress [29]. Taken together, these findings suggest a role for sex differences in genome function and regulation in sex-specific etiologies of stress-related disorders [30].

Although allele frequencies do not differ between males and females across the autosomes [31], GWAS sufficiently powered to allow stratification by sex has demonstrated the heterogeneity of genetic effects between males and females in association with complex traits [30]. Genetic variants may indeed show sex bias in their regulation of gene expression, supported by identified autosomal sex-biased *cis*-expression quantitative trait loci (**eQTLs**) in whole blood [21, 32]. Thus, in addition to regulation across the genome by gonadal hormones, there may also be sex-specific influences of genetic variants on downstream epigenetic and regulatory elements. Targeting these sex differences in genetic regulation of stress pathways, in particular, may elucidate sex-specific pathways of risk for psychiatric disorders.

Previously, we explored genetic variants that regulate the **GR-response**, defined as the immediate transcriptional response to glucocorticoids in humans, in our design via administration of dexamethasone, a selective agonist for GR [33]. By quantifying gene expression in peripheral blood at baseline and three hours post dexamethasone administration, we reported eQTLs which modulate the transcriptome response to GR-activation in men. The eQTL SNPs (eSNPs) were shown to be enriched among genetic variants associated with schizophrenia as well as MDD and to predict amygdala reactivity to threat [33] as well as neurovascular-coupling related features of the neural stress response [34]. The transcripts regulated by these variants form tight co-expression networks. Using an animal model of exposure to adversity across development [35], we observed that different combinations of early and adult environments (supportive vs. stressful) substantially affect the co-expression structure of these networks in a highly brain region-specific manner [36]. However, this set of eQTLs and regulated transcripts was identified in a male-only cohort.

Given the above-described sex differences in the stress-response as well as in the prevalence and manifestation of psychiatric disorders, we conducted a sex-stratified analysis of genetic regulation of the transcriptional response to GR-activation in peripheral blood cells. We found that while transcripts regulated by GR-activation were largely overlapping in males and females, genetic variants moderating these GR-induced transcriptional changes (GR-eQTLs) were mainly identified in only females or males, suggesting that distinct genetic features moderate the transcriptional response to GR-activation in the two sexes. The transcripts regulated by GR-eQTLs (etranscripts) were enriched among sex-stratified transcriptional signatures of MDD in post-mortem brain tissue [29]. Sex-stratified GR-eQTLs were enriched in GWAS signals for MDD. Transcriptional sensitive genetic profile scores derived from sex-stratified GR-eQTLs also predicted depression and depressive symptoms in an adolescent cohort in a sex-specific manner. Our results underline the importance of sex-stratified analyses in stress-induced gene-regulation for a better understanding of stress-related psychiatric disorders.

## Results

**Fig. 1:**
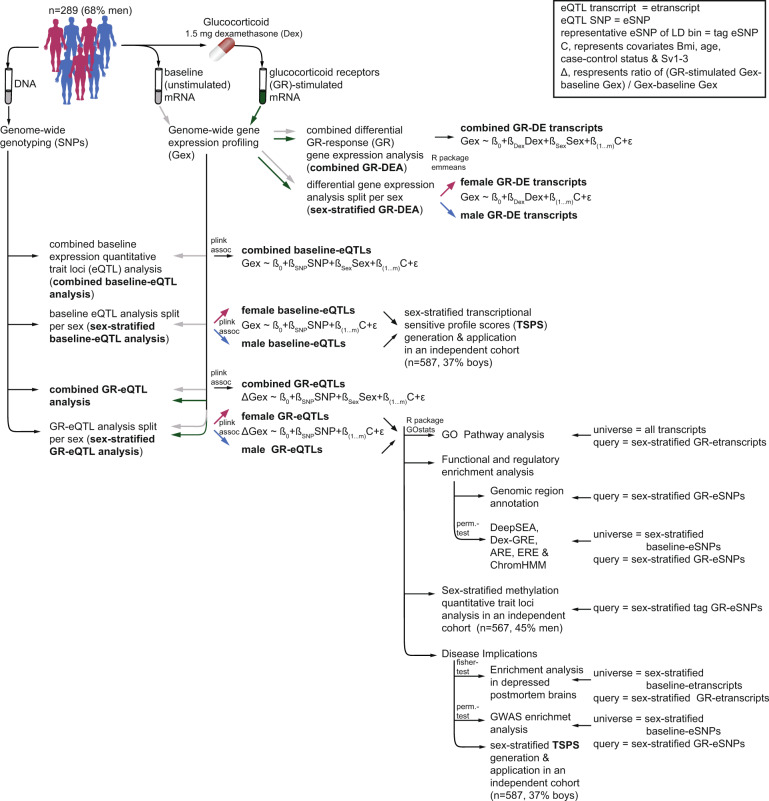
Study Design and Work Flow. Flow of data collection and statistical analyses: Genome-wide genotyping and gene expression profiling were used to examine differential GR-response gene expression and expression quantitative trait loci in (1) combined and (2) sex-stratified analyses. Results were carried forward for functional interrogation and linkage to disease.

Whole blood samples from 289 individuals (93 females [48 patients with depression and 45 healthy controls] and 196 males [81 patients with depression and 115 controls]) recruited at the Max Planck Institute of Psychiatry (MPIP) were analyzed for gene expression levels at baseline and three hours post stimulation by the selective GR-agonist dexamethasone (see Table 1 for description). 11,994 transcripts were entered into the statistical analysis. Additionally, all samples were genotyped, with a total of 3.9 Million SNPs available for analysis. All analyses were conducted only on autosomes to allow comparison between males and females and controlled for age, case-control status, BMI, and cellular heterogeneity using surrogate variables (*n* = 3, see Supplementary Fig. 1). Figure 1 displays an overview of the data analysis and results outlined below.

**Table 1:** Clinical characteristics. For continuous data the mean ± standard error and for categorical data the categories separated by dashes are given for females and males.

	MPIP cohort		LMUC cohort		recMDD cohort	
Sex	males	females	males	females	males	females
N	196	93	201	383	255	312
Age	42.65 ± 13.7	42.95 ± 14.6	14.5 ± 2.3	15.5 ± 1.8	46.53 ± 13.9	47.0 ± 13.8
BMI	25.4 ± 3.3	23.9 ± 5.1	NA	NA	24.7 ± 3.1	24 ± 4.5
N control/cases	115/81	45/48	115/86	235/148	78/177	114/198

### GR-stimulated gene expression: comparison of males and females

**Fig. 2:**
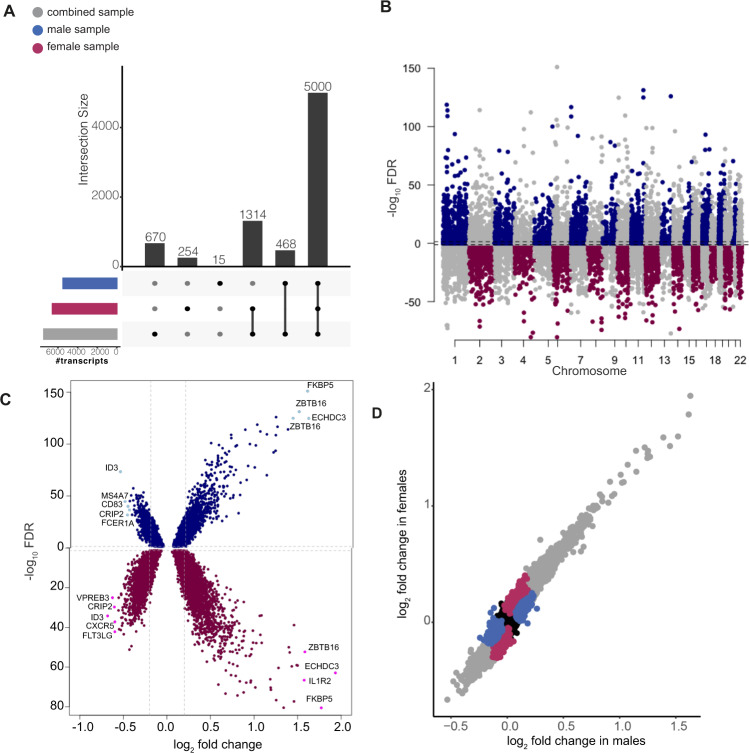
Differential GR-response gene expression analysis results. Differential GR-response gene expression analysis (GR-DEA): **A** Upset plot displaying the overlapping significant transcripts identified in combined and sex-stratified GR-DEA. The majority of transcripts were found in both the combined and stratified analyses independently. **B** Miami plot of results across 11,994 autosomal transcripts. Dashed lines indicate significance cut-off at an FDR of 5%. 6,568 GR-DE transcripts were significantly differentially regulated in females (*n* = 93 individuals; bottom panel) and 5483 GR-DE transcripts in males (*n* = 196 individuals; top panel). **C** Volcano plot of log2 fold change (*x-*axis) by −log_10_*FDR*. Upper panel showing male GR-DE transcripts at an FDR of 0.05 with FCs ranging from 0.68 to 3.06. Lower panel showing female GR-DE transcripts with FCs ranging from 0.62 to 3.82. **D** Scatterplot showing the difference in gene expression between post dexamethasone and baseline for males (*y*-axis) and females (*x*-axis) colored by identification in combined analysis (*n* = 5000 transcripts), females (*n* = 1568), males (*n* = 483), and neither females or males (*n* = 4943). Significant results, whether supported in the combined analysis or limited to sex-stratified analyses, are mainly limited to the upper right and lower left quadrants, supporting consistent effect directions between males and females.

First, we assessed the main effects of dexamethasone on gene transcription in a combined differential GR-response gene expression analysis (combined GR-DEA) in all participants controlling for sex (Fig. 1). These results were then compared to differential gene expression analyses stratified by sex (sex-stratified GR-DEA), as well as a differential gene expression analysis testing the effect of sex on GR-stimulated change in gene expression (see Supplementary Results and Supplementary Table 1). The combined GR-DEA identified 7462 out of 11,994 autosomal transcripts to be significantly differentially expressed at an *FDR* of 0.05, and 2352 transcripts (31.5%) to surpass an absolute log_2_ fold change (FC) threshold of 0.2 (see Supplementary Table 2). The majority of transcripts found to be regulated by dexamethasone in the combined GR-DEA were also identified in the sex-stratified GR-DEA, with few additional transcripts emerging (*n* = 253 in females and *n* = 15 in males; Fig. 2A). Next, we assessed the consistency of the magnitude and direction of GR-DE changes across males and females (Fig. 2B, C, Supplementary Table 2). Overall, larger log FCs were found in females (Fig. 2D and Supplementary Fig. 2). Further analyses supported that effects sizes, rather than direction, were moderated by sex, with consistent effect directions found in males and females (Fig. 2C, D, see Supplemental Results). Sex-stratified GR-DEA effects were likely not driven by differences in dexamethasone serum levels. At the timepoint of the second blood draw, no differences in dexamethasone levels were observed between sexes in a subset of 162 males and 68 females (mean ln dexamethasone level = 2 ± 0.25 in males and 1.92 ± 0.93 in females, *p* value = 0.46). Thus, we conclude that sex differences in GR-response are largely due to the magnitude of the transcriptome change rather than the direction of the effect.

### Sex differences in genetic regulation of GR-response

We next investigated sex differences in the genetic regulation of the transcriptional GR response. We focused on *cis*-eQTLs, which were defined as associations between SNPs and transcripts within a 1 Mb window. C*is*-eQTL analyses were performed to identify baseline eQTLs (Supplementary Table 3, eSNPs significantly related to gene expression in unstimulated mRNA) and GR-eQTLs (eSNPs significantly related to the change in gene expression after GR stimulation). These analyses were carried out again in the combined sample (combined baseline-eQTL analysis and combined GR-eQTL analysis) and stratified by sex (Supplementary Tables 4 and 5, sex-stratified baseline-eQTL analysis and sex-stratified GR-eQTL analysis). Although a cohort was not available to validate sex-stratified GR-eQTLs, we used publicly available data to validate sex-stratified baseline-eQTLs. We focused on the overlap of *cis* GR-eQTL effects in the sex-stratified analysis (i.e., common combinations of eSNPs and etranscripts), and the consistency of effect sizes and directions between males and females.

The combined GR-eQTL analysis identified 10,398 significant GR-eQTLs after multiple test correction, involving 717 etranscripts and 10,078 eSNPs. The 10,078 unique GR-eSNPs can be summarized into 747 uncorrelated GR-eSNP bins, i.e. sets of SNPs in linkage disequilibrium (LD) represented by a tag eSNP (see “Methods”^33^). These 747 tag GR-eSNP bins correspond to 804 GR-eQTL bins, i.e., eSNP bin–probe combinations, with some tag eSNPs associated with the expression of more than one transcript and are listed in Supplementary Table 6.

**Fig. 3:**
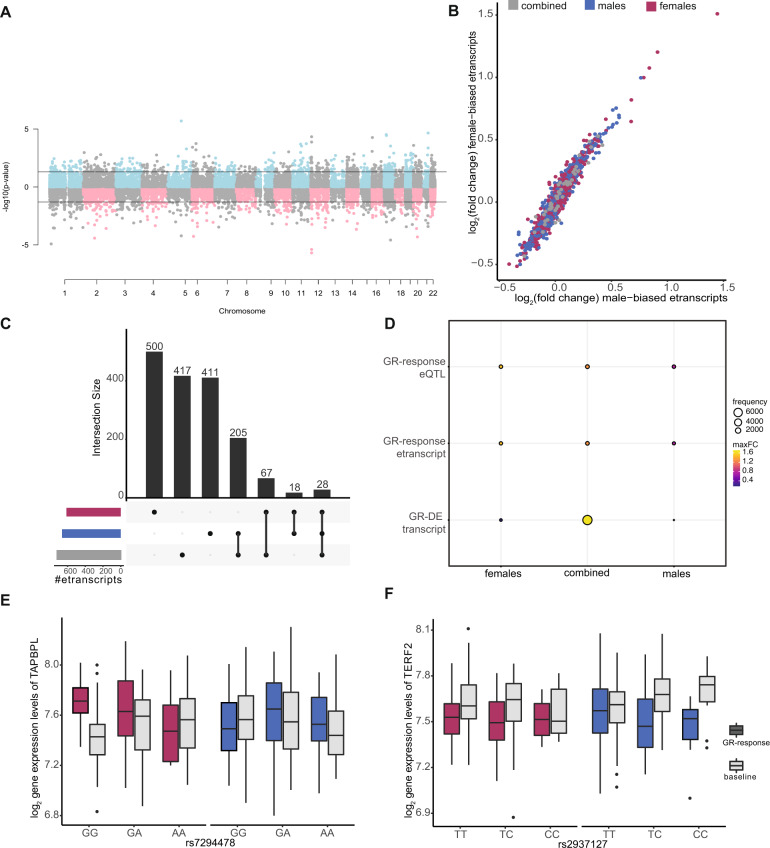
GR-response *cis*-eQTL analyses results. GR-response *cis*-eQTL analyses: **A** Miami plot of eQTL results. Only the best eQTL per etranscript is plotted. Dashed lines indicate significance cut-off at an FDR of 5%. **B** Mean log2 fold changes between post dexamethasone and baseline colored by identification of etranscripts in combined analysis (*n* = 46 transcripts), females (*n* = 567) or males (*n* = 616). The effects of the etranscripts for male and females were similar (Wilcoxon *p-*value = 0.7). **C** Upset plot displaying the overlapping significant GR-response etranscripts identified in combined analysis, males, and females. The majority of these transcripts were specific to females (91%, *n* = 193), whereas 68 (59%) transcripts were specific to males and 74 (57%) transcripts were found in the combined eQTL analysis. **D** Balloon plot showing the frequency of transcripts found in (1) females but not the combined analysis, (2) the combined analysis, and (3) males but not the combined analysis, across GR-DE transcripts, etranscripts, and etranscript-eSNP pairs. In the GR-DE analysis, the majority of transcripts are identified in the combined analysis, whereas etranscripts and eSNP pairs (eQTLs) show more of an even distribution across females, combined, and males. Maximum fold changes were higher in female etranscripts relative to males. **E**, **F** Boxplots of overlapping significant GR-DE transcripts and etranscripts. Gene expression is stratified by eSNP and shown for females and males. **E** Tag eSNP rs7294478 is located in an intron of *C1RL-AS1* on chromosome 12. However, the eQTL effect was observed only in females on *TAPBPL* expression, which is located over 700 kb downstream. *TAPBPL* itself is one of the significant DR-DE genes identified in females (FDR = 0.00068 vs. FDR = 1 in males). **F** The intronic tag eSNP rs2937127 demonstrates no effect in females, while in males the minor allele was associated with a down regulation of *TERF2* gene expression (FDR = 0.04). *TERF2* is located approximately 470 kb upstream of the tag eSNP, which is positioned in the gene *WWP2*.

The sex-stratified GR-eQTL analysis (Fig. 3A) again indicated that effect directions were consistent between males and females (Fig. 3B). In females, GR eQTLs were found for 648 eQTL bins comprising 613 etranscripts and 601 tag eSNP (Supplementary Table 7). Slightly more eQTLs were identified in males with 705 eQTL bins involving 662 etranscripts and 668 tag eSNPs (Supplementary Table 8). By overlapping the female and male stratified results with the combined GR-eQTL analysis, we show that 34% of the male GR-response etranscripts (*n* = 233) and 16% of the female GR-response etranscripts (*n* = 95) were identified as etranscripts by the combined model (Fig. 3C). Thus, in contrast to the GR-DEA results, the sets of etranscripts are largely non-overlapping (Fig. 3D and Supplementary Figs. 3, 4**)**.

An example of a female GR-eQTL compared to males and to the combined sample is displayed in Fig. 3E, F. Approximately 50% of etranscripts identified in the sex-stratified GR-eQTL analysis were also identified as sex-stratified GR-DEA transcripts (Fig. 3D), with female etranscripts exhibiting larger log_2_FCs relative to males (see Supplemental Results**)**. We next compared enrichment of biological functions for GR etranscripts between males and females. Female etranscripts were enriched for regulation of natural killer cell-mediated immunity and male etranscripts were enriched for regulation of cyclin-dependent protein kinase activity, positive regulation of extrinsic apoptotic signaling pathway, peptide metabolic processes, and other functions (see Supplementary Table 9). Additionally, we validated the majority (86% male baseline eQTLs and 84% female baseline eQTLs) of baseline eQTLs in publicly available data (see Supplementary Results).

### Functional and regulatory context of sex-stratified GR-eSNPs

**Fig. 4:**
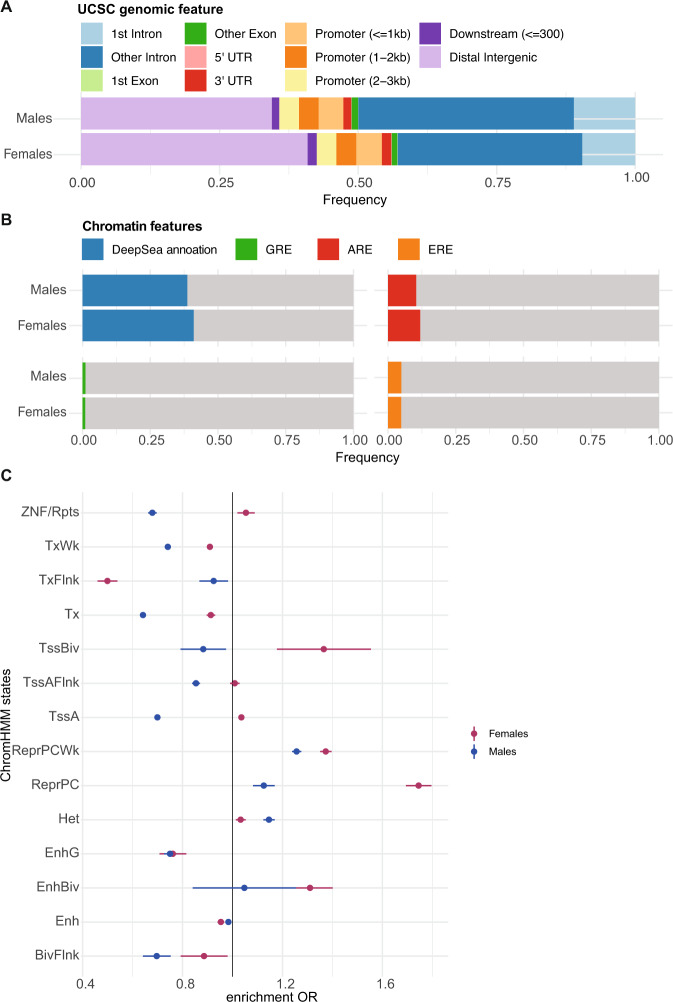
Sex stratified GR-response *cis*-eQTL Functional Results. Sex stratified GR-response *cis-*eQTLs and chromatin annotation: **A** Annotation of the genomic regions in which eSNPs are located. **B** Bar plots showing the overlap of GR-eSNPs and DeepSea annotations, Remap transcript factors (AR and ER) and Encode GR-Chip peaks. **C** Enrichment results for GR-response tag eSNPs and predicted ChromHMM states for sex-stratified tag eSNPs.

We next characterized the identified GR-eSNPs (unpruned) in terms of genomic location, regulatory features, and enrichment for sex hormone response elements. GR-eSNPs for females were significantly more likely to be located in distal intergenic regions (40.9%) compared to male GR-eSNPs (34.4%), see Fig. 4A**(**fisher exact *p-*value = 1.4 × 10^−14^). Male GR-eSNPs were significantly more likely to cluster in intronic regions (50% vs. 42.9% in first or other introns for male and female GR-eSNPs, respectively (fisher exact *p-*value = 3.4×10^−16^).

As eQTLs have previously been associated with regulatory regions [37], we quantified enrichments of male and female GR-eSNP sets for regulatory features. For all tests, enrichment for sex-stratified GR-eSNPs were tested for significant enrichment compared to sex-stratified baseline eSNPs set as background to ensure sex-stratified effects were specific to eSNP regulation of the GR-response, specifically. Thus, male and female GR-eSNPs had to show significant enrichment relative to male and female baseline eSNPs. First, we used DeepSEA, a deep neural network pretrained with DNase-seq and ChIP-seq data from the ENCODE project, to predict the likelihood that sex-stratified GR-eSNPs exert regulatory effects on chromatin features. We found 8.4% of the female GR-eSNPs (*n* = 500) with significantly overlapping DeepSEA features (*e-*value < 0.01) and 10.7% of the male GR-eSNPs (*n* = 851), contained DeepSEA features (Fig. 4B). Additionally, using GRE ChIP-Seq peaks from ENCODE lymphoblastoid cell lines treated with dexamethasone, we observed significant overlap within GR-binding sites (GREs) for female GR-eSNPs (*n* = 58 out of 5586 eSNPs, enrichment *p-*value = 0.022, *OR* = 1.46, Fig. 4B), but not male GR-eSNPs.

To determine if the sex-stratified GR-eSNPs are more likely to be located within sex hormone responsive regulatory elements, we calculated the number of eSNPs that are located within androgen response elements (AREs) and estrogen response elements (EREs), using data from Remap (see “Methods”). Of all 5,586 female GR-eSNPs, 4.89% (*n* = 273, Fig. 4B) were located within EREs and 11.94 % (*n* = 667, Fig. 4B) in AREs. For the 7771 male GR-eSNPs, 4.95% (*n* = 382, Fig. 4B) and 10.38% (*n* = 807, Fig. 4B) were located within EREs and AREs, respectively. Enrichments for AREs and EREs were not statistically significant above sex-stratified baseline eQTLs. These results suggest that sex-stratified GR-eSNPs may potentially be independent of the direct influence of sex hormones, in accordance with previous results [30, 38].

Sex-stratified GR-eSNPS were additionally tested for enrichment for hormone-related transcription factors (TFs) including ESR1, AR, and NR3C1 using Remap. Although both male and female sex-stratified GR-eSNPs and sex-stratified baseline eSNPs demonstrated significant enrichments across these TFs, the sex-stratified GR-eSNPs were not significantly enriched relative to sex-stratified baseline eSNPs. Testing the full remap database, we found significant enrichment of EZH2 and NR5A2 for female GR-eSNPs above baseline eSNPs, and significant enrichment of SND1 and EZH2 for male GR-eSNPs above baseline eSNPs.

Using the 15-state ChromHMM annotation of the Roadmap Epigenomics project [39], we observed that both female and male GR-eSNPs were enriched within repressed polycomb and bivalent enhancers across the tissue group of blood and T-cells (*n* = 14 cell lines), see Fig. 4C. Female GR-eSNPs were enriched in ZNF genes and repeats, bivalent and poised transcription start sites (Tss), and active Tss (TssA and TssAFlnk), while male GR-eSNPs were depleted in Tss (Fig. 4C). For the individual blood cell lines and enrichment *p-*values, see Supplementary Fig. 5. All results were consistent whether using all eSNPs, or limiting the analysis to tag eSNPs, suggesting that results were not dependent on the structure of eSNPs in LD.

### Epigenetic modifications of sex-stratified GR eSNPs

As regulatory effects of sex-stratified GR-eSNPs may also act at the level of the epigenome, we explored links between sex-stratified GR-eSNPs and DNA methylation levels at baseline in an independent sample (recMDD cohort, see “Methods”) of 312 females and 255 males. We first performed sex-stratified methylation QTL (meQTL) analyses and identified 10,832,433 meQTLs in males comprising 163,238 CpGs and 2,94 million SNPs (5% FDR). Additionally, we found 12,691,324 meQTLs in females comprising 162,773 CpGs and 3,16 Mio SNPs at an FDR of 5% with 51.1% CpGs (*n* = 83,228) and 74.2% meQTL SNPs (meSNPs; *n* = 2.343.464) in common with the CpG identified in males. Next, we quantified the number of sex-stratified GR-eSNP that are also significant meSNPs. Approximately half of both the female and male tag GR-eSNPs were meSNPs, i.e., 317 out of 601 female tag GR-eSNPs and 319 out of 668 male GR tag eSNPs (Supplementary Fig. 6A–C). Thus, half of the sex-stratified eSNPs had additional associations with DNAm patterns.

### Disease Implications: sex-stratified GR-eQTLs predict depression and depressive symptoms

The potential disease relevance of the sex-stratified GR eQTLs was explored at three levels: enrichment in depression-related DE in human postmortem brain tissue, enrichment in GWAS associations for psychiatric disorders and traits and association of genetic profile scores weighted by sex-specific etranscript regulation.

#### Postmortem gene expression in major depression

**Fig. 5:**
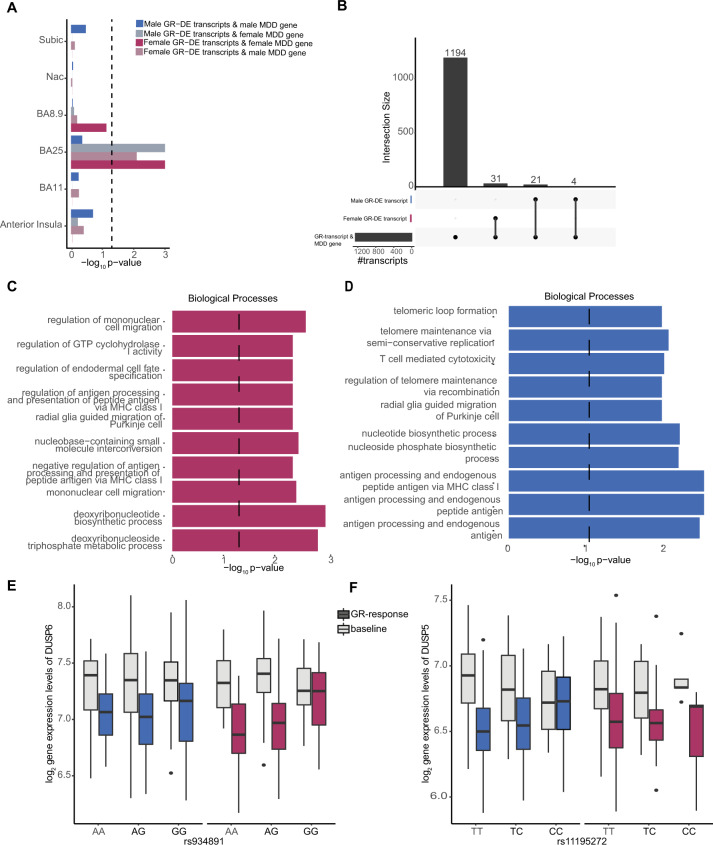
Sex-stratified GR-response etranscripts in the Brain. Sex-stratified GR-response etranscripts represented in MDD transcriptional patterns: **A** Bar plot showing the significance of GR-etranscripts for enrichment in MDD transcriptional profiles in six brain regions. Both male and female GR-etranscripts were tested against male and female MDD transcriptional profiles. The black line indicates significance cut-off at a *p-*value of 0.05**. B** Upset plot displaying the overlapping significant sex-stratified GR-response etranscripts with BA25 MDD-related transcripts. **C** GO enrichment results for female etranscripts overlapping with BA25 MDD-related transcripts. **D** GO enrichment results for male etranscripts overlapping with BA25 MDD-related transcripts. **E** *DUSP6* example showing gene expression at baseline and post dexamethasone across genotypes of tag eSNP rs934891 for males and females (female *FDR* = 0.049). **F** *DUSP5* example showing gene expression at baseline and post dexamethasone across genotypes of tag eSNP rs11195272 for males (male *FDR* = 0.046) and females.

We next explored whether sex-stratified GR-etranscripts and eSNPs were represented within previous findings on genetic risks and underpinnings of psychiatric disorders. First, sex-stratified GR-etranscripts (relative to sex-stratified baseline etranscripts) from blood were mapped to sex-stratified transcriptional differences in the brain in association with MDD [29]. Male GR-etranscripts were significantly enriched (*FDR* < 5%) in Brodmann area (BA) 25 in female MDD genes, and female GR-etranscripts were enriched in BA25 in both male and female MDD genes, a critical area for mood disorders, targeted by deep brain stimulation in the treatment of depression [40], see Fig. 5A. Neither male or female GR-etranscripts were significantly enriched in other brain regions.

Sex-stratified GR-etranscripts overlapping with female MDD-related BA25 transcripts included 37 female GR-etranscripts and 27 male GR-etranscripts (with 4 common GR-etranscripts between males and females, Fig. 5B). We tested whether these sex-stratified GR-etranscripts exhibited functional pathway differences between males and females. Female overlapping GR-etranscripts were significantly enriched for deoxyribonucleotide biosynthetic process and deoxyribonucleotide triphosphate metabolic process. Male overlapping GR-etranscripts were enriched for antigen processing and presentation of endogenous peptide antigen (OR = 64.76, nominal *p-*value = 0.0007) among other antigen processing functions, and nucleotide biosynthetic process (OR = 9.03, nominal *p-*value = 0.002) (Fig. 5C, D). Interestingly, Dual Specificity Protein Phosphatase 6 (*DUSP6)* was represented among female GR-etranscripts, and *DUSP5* within male GR-etranscripts, both members of an enzyme subfamily of dual-specificity MAP kinase phosphatases which are conserved in domain structure. *DUSP6*, in particular, was identified as a driving hub in MDD-related transcriptional networks [29] and is involved in brain-related functions via inactivation of ERK pathways. Labonté and colleagues found that *DUSP6* was downregulated in female MDD subjects in BA25, and this pattern of downregulation was further supported by a mouse model of MDD in chronically stressed female mice. Although we found transcriptional effects in *DUSP6* to be common in males and females in response to GR activation, the eSNP effects were specific to females (Fig. 5E), highlighting a sex specific mechanism regulating a common, downstream physiological pattern. *DUSP5*, similarly involved in ERK signaling in the brain, was also downregulated by GR activation in males and females in our GR-DE analysis, but with a specific eSNP effect for males (Fig. 5F).

#### GWAS for psychiatric disorders and traits

**Fig. 6:**
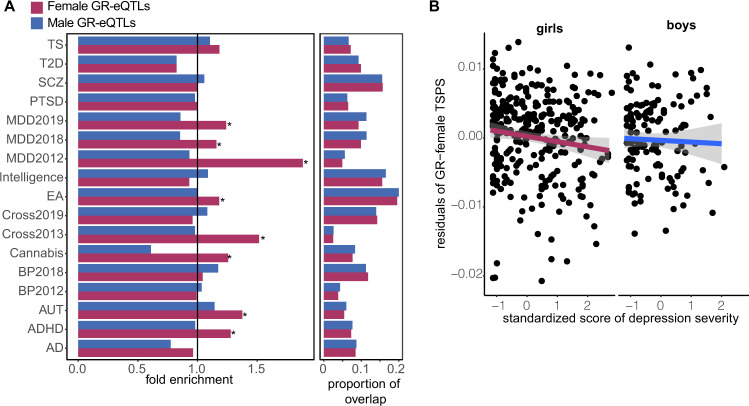
Sex-stratified GR-response eSNP associations with psychiatric disorders. **A** Bar plot of enrichment results for GR-response tag eSNPs and GWAS SNPs. The black indicates a fold enrichment at 1 and a star indicates a permutation-based FDR < 0.05. AD = Alzheimer’s disease, ADHD = attention-deficit/hyperactivity disorder, AUT = autism spectrum disorders, BP = bipolar disorder, Cross = cross disorder analysis, EA = educational attainment, MDD = major depressive disorder, PTSD = post-traumatic stress disorder, SCZ = schizophrenia, T2D = diabetes type 2, TS = Tourette syndrome. **B** Association between residualized female TSPSs and standardized scores of severity of depressive symptoms computed in LMU cohort (girls: β = −7.98 × 10^−4^, SE = 4.04 × 10^−4^, *p-*value = 0.0496; boys: β = 1.12 × 10^−3^, SE = 8^.3^ × 10^−4^, *p-*value = 0.18).

To extend these results, we tested whether sex-stratified GR-eSNPs were overrepresented among GWAS SNPs associated with psychiatric disorders using large-scale GWAS results of the Psychiatric Genomics Consortium (PGC), relative to sex-stratified baseline eSNPs. All enrichments were independent of LD as we used the top-associated SNP of the clumping procedure (i.e., the tag SNP). We detected a significant enrichment of female GR-eSNPs (*n* = 598 tag eSNPs) compared to female baseline eSNPs (*n* = 1,074 tag eSNP) with SNPs at a nominal GWAS *p-*value cutoff associated with MDD (fold enrichments = 1.15–1.88, permutation-based *FDRs* < 0.05, educational attainment (fold enrichment = 1.18, permutation-based *FDR* = 0.003), autism spectrum disorder (fold enrichment = 1.38, permutation-based FDR < 0.001), attention-deficit/hyperactivity disorder (fold enrichment = 1.28, permutation-based *FDR* = 0.013), cannabis intake (fold enrichment = 1.26, permutation-based *FDR* = 0.012) and the cross-disorder analysis 2013 (fold enrichment = 1.5, permutation-based FDR = 0.007), see Fig. 6A. For male GR-eSNPs we did not identify an enrichment over male baseline eSNPs. In summary, female eSNPs regulating the GR response, but not male eSNPS, were significantly enriched in SNPs identified in relation to psychiatric disorders in large-scale GWAS studies.

#### Sex-specific genetic profile scores

Given the highly distinct sets of genetic variants regulating the GR-response in males and females, we assessed whether the genetic variants of sex-stratified GR-eQTLs would be cumulatively associated with sex-stratified sensitivity for psychiatric disorders. Transcriptional sensitive profile scores (TSPS) were calculated by summation of the GR-eQTL effects. The ‘sensitive’ allele is defined as the allele with the highest absolute eQTL effect, regardless of effect direction, such that a higher TSPS represents elevated sensitivity for a GR-moderated transcriptional response. We tested whether TSPS based on sex-stratified eSNPs (sex-stratified TSPS) were associated with depression and depressive symptoms. We applied sex-stratified TSPSs to a clinical cohort comprising 350 Caucasian children and adolescents 7–18 years old with a current diagnosis or history of MDD (67% girls) and 234 healthy control subjects (ages 7–18 years old) with no history of a psychiatric disorders (63% girls, see “Methods”). Female TSPS significantly predicted case control status for depression in girls (*p-*value = 0.0256, see Fig. 6B), explaining 2.3% of the variance in depression. Both the male and female TSPS significantly predicted specific depressive symptoms in the respective sex (*p-*values < 0.05, see Supplementary Fig. 7). For instance, female TSPS significantly related to irritability, loss of satisfaction, agitation, crying, suicidal ideation, feelings of failure, and self–dislike, whereas male TSPS significantly related to changes in appetite, self-deprecation, anhedonia, and loss of interest. Both TSPSs significantly relate to worthlessness. Overall, female depressive symptoms were more self-directed or brooding than male symptoms.

Taken together, we found connections between sex-stratified GR-eSNPs and (1) transcriptional patterns in the brain in relation to MDD in women, (2) SNPs associated with psychiatric disorders, and (3) depression status and symptoms in a developmental cohort. Female eSNPs, in particular, were enriched in SNPs identified in psychiatric disorders, and as a cumulative score, were predictive of case-control status. Thus, sex-stratified GR-eSNPs, identified as regulating the GR transcriptional response in our sex-stratified analysis, may have relevance for the etiology of psychiatric disorders and implicate biological risk for their development in response to stress exposure.

## Discussion

Sexual dimorphism in the stress response is well established, but how these sex differences are genetically regulated and linked to sex-specific risk for psychiatric conditions are unknown. Here, we explored potential sex differences in regulation of the stress response by comparing GC-induced changes in gene transcription and *cis* genetic regulation of these changes in males and females. We find that sex differences in the transcriptomic GR- response are largely due to females demonstrating stronger effects of GR activation in terms of up and down regulation of transcripts, rather than differences in the direction of effects. However, the genetic regulation of the transcriptomic GR-response was highly disparate between sexes, with males and females demonstrating distinct sets of genetic variants corresponding to distinct patterns of regulatory features. Genes differentially expressed in response to GR activation in blood in males and females also demonstrated sex-specific transcriptional patterns in postmortem brain of female patients with depression, and female GR-eQTLs were enriched among SNPs identified in large scale GWAS studies in relation to psychiatric disorders. Moreover, sex-stratified TSPSs created from sex-stratified GR-eSNPs predicted depression status and depressive symptoms in a clinical cohort of children and adolescents. Taken together, these findings have implications for identifying genetic sensitivity factors for males and females, corresponding to sex-specific biological susceptibility to stress exposure and stress-related psychiatric disorders.

Male and female GR-eQTLs could emerge due to direct genetic effects within the binding sites of GR, as well as due to epigenetic mechanisms at the level of chromatin [41]. To explore epigenetic mechanisms in relation to sex-stratified GR-eQTLs, we performed an integrative analysis of epigenetic states, including overlap of eSNPs with GR and sex hormone-binding sites and linkage to sex-stratified SNP effects on DNAm (meQTLs). For both female and male GR-eQTLs, we found enrichment for regulatory chromatin features, but with sex-specific enrichments. Male and female GR-eQTLs which overlapped with sex hormone response elements were not enriched above male and female baseline eQTLs, suggesting that male and female GR genetic regulation may be independent of direct influences of sex hormones. We found that a substantial proportion (about half) of etranscripts regulated by sex-stratified GR-eQTLs were linked to sex-stratified meQTLs. Together, these results suggest that male and female GR-eQTLs have distinct downstream regulatory effects upon GR pathways and are also associated with sex differences in DNA methylation status, which may be important for sex differences in gene expression. Further, these regulatory effects appear to be, at least in part, independent of circulating sex hormones.

Previously, the study of biological differences between males and females largely targeted brain organization and regulation by sex hormones. More recently, attention is being paid to growing evidence in favor of genetic and epigenetic regulation of sexual dimorphism in behavior [42]. By activating GR to directly assess genetic regulation of the stress response in males and females separately, our results add to a growing body of literature highlighting sex differences in gene expression and genetic regulation [23, 29, 38, 43, 44]. In contrast to much of the work on the genomics of sex differences, we find that males and females differ in genetic regulation outside of the X and Y chromosomes. Thus, this work suggests that the genetic regulation of sex differences in stress responding extends to the autosomes, and highlights the need for further work to understand the sex-specific genetic and epigenetic architecture underlying susceptibility to stress-related disorders.

The sex differences in genetic regulation of the blood transcriptome response to stress reported here are consistent with growing work in animal models and humans on sex differences in the regulation of genes, and at least for animals, in response to manipulated environmental stress. Animal models have shown sex-specific divergence in transcriptional patterns following chronic and acute stressors in the brain [26, 27, 29] as well as in specific GR-system moderators in blood [45]. In humans, studies on sex-related divergence in gene regulation following events or stress are extremely limited, with robust results on sex differences in genetic regulation of gene expression at baseline across tissues only recently reported [46]. However, adding our results do build upon studies reporting sex specific transcriptional signatures of stress-related disorders, including MDD and PTSD [29, 47–50].

Male and female etranscripts that were regulated by GR-eQTLs were found to be enriched for genes previously reported as sex-specific MDD transcriptional signatures in the brain. For these sets of significantly enriched genes identified in blood, their representation in the brain was not specific to males or females, despite the fact that these neural transcriptional signatures showed strong sex specificity in postmortem brain [29]. These results echo additional results presented in Labonte et al., namely, that although the transcriptional correlates of MDD in the brain were highly disparate between males and females, the downstream pathways of stress susceptibility converged. Interestingly, the enrichments were restricted to DE transcripts in BA25, the subgenual cingulate region, a brain area implicated in the pathophysiology of major depression and a target for deep brain stimulation as treatment for therapy resistant forms of this disease [51].

We have previously shown that GR-eQTLs in males are enriched among genetic variants associated with risk for psychiatric disorders, including MDD and SCZ [33]. The female GR-eQTLs we identified here were enriched for SNPs associated with MDD, EA, cannabis use, AUT, ADHD in large-scale GWAS as well as cross disorder psychiatric risk [52–54]. The selective enrichment of female GR-eQTLs in GWAS is interesting, as not all of the above disorders have a higher prevalence in girls or women. This would suggest, as also highlighted above, that sex-stratified GR-eQTLs target common pathways of risk, and emergence of disease is driven by a number of additional factors. A limitation of our enrichment analyses is that current GWAS mainly combine data from both sexes, even though a previous *post hoc* analysis of existing GWAS studies identified numerous significant loci that were driven by one sex or the other [55] and another study identified genetic variants associated with MDD status in females only [56]. Our results and these studies highlight that large-scale studies aimed at genetic discovery may benefit from modeling males and females separately.

Large-scale GWAS have been used to derive polygenic risk scores, weighted by association strength to predict disease risk or better understand correlated biological features and comorbidities across disorders. However, these scores are limited by the fact that the underlying GWAS rely on heterogenous samples and imprecise measurement of complex phenotypes [57]. Here, by manipulating the biological system of interest, we were able to preselect SNPs based on functional regulation. We weighted these SNPs by transcriptional changes in response to GR-activation by dexamethasone, a direct gauge of the biological stress response shaped by an individual’s history of stress exposure. Thus, this design allows the isolation of genetic variability relevant to biological stress responding shaped across developmental time, and regardless of the diversity of individuals’ environmental histories and idiosyncratic responses to stressful events.

These genetic sensitivity scores indeed demonstrated relevance to stress-related disorders. TSPS scores predicted depression status as well as symptoms in a sex-specific manner. Thus, both the genetic etiology, and the relations of these genetic sensitivity scores to MDD symptoms are specific to sex. Across sex-biased symptomology, higher scores of GR-eQTL dosage associated with larger biological responses to GR activation were associated with lower levels of depressive symptoms and status. This is in line with data from stress and trauma research, showing that a blunted cortisol response is associated with higher risk for subsequent psychiatric disorders.

It is important to acknowledge a number of limitations to this study. First, our sample size, although considerably expanded relative to our previous report [33], is still small for detecting small differences between males and females in the genetic regulation of the GR-response and was imbalanced between males and females. Although GR activation by dexamethasone offers a substantial biological effect at the level of the transcriptome, replication of our results in an independent cohort is necessary. However, bootstrapping analysis indicated overall robustness of our finding (see “Results”). In addition, the majority of the sex-stratified baseline-eQTLs were also significant in public data, and thus we were well powered enough to replicate previous blood eQTL findings. Second, we were unable to control for timing of the menstrual cycle and the use of birth control in women. Although this should be addressed in a replication, accounting for surrogate variables reflecting cell-type proportions in our data should ameliorate any effects of this unwanted biological variation.

To the best of our knowledge, we are the first to report sex-stratified effects of GR activation in terms of differential gene expression in human blood. Moreover, this is the first study to identify male and female specific GR-eQTLs. In contrast to previous studies of biological sex differences in humans that often focus on sex chromosomes, we find significant and robust sex differences in terms of autosomal genetic variants in their regulation of the stress response with relevance to stress-related diseases. We report that these sex differences, both at the level of differential expression and genetic regulation of the GR-response, are large and robust enough that they emerge even in combined-sex models that control for sex. These findings highlight the need for careful examination of sex differences in the study of genetic risks and the biological substrates of stress-related disorders.

## Methods and materials

### Study samples

#### MPIP cohort

Participants consisted of 289 Caucasian individuals of the Max Planck Institute of Psychiatry (MPIP), 93 women and 196 men. Sex was defined by the sex chromosomes (X and Y), which is distinct from the biopsychosocial concept of gender [58]. Of the participants, 129 (81 men, 48 women) were being treated for MDD treated at the MPIP’s hospital in Munich and the remaining were 160 (115 men, 45 women) were healthy controls with no history of a depressive disorder, see Table 1. Recruitment strategies and further characterization of the MPIP cohort have been described previously [33], in a previous analysis of *n* = 160 males. Baseline whole blood samples were obtained at 6 pm after 2 h of fasting and abstention from coffee and physical activity. Subjects then received 1.5 mg oral dexamethasone and a second blood draw was performed at 9 pm three hours after dexamethasone ingestion. Plasma dexamethasone concentrations were assessed in serum samples drawn at 9 pm using Liquid chromatography-tandem mass spectrometry on API4000 (AB Sciex).

#### LMU cohort

The clinical LMU cohort consists if 584 Caucasian children and adolescents (ages 7–18 years old) recruited from two child and adolescent clinics in Munich: 350 cases with a current diagnosis or history of major depression and 234 healthy control subjects with no history of a psychiatric disorder. The presence or absence of depression was determined by a well-established diagnostic interview [59]. Further characterization of the cohort and psychometric measures are described in [60] and Table 1. To assess the severity of depressive symptoms, the Children’s Depression Inventory (CDI) was administered to youths ≤12 years old, and the Beck Depression Inventory-II (BDI-II) was administered to participants >12 years old. Scores from the CDI and the BDI-II were standardized using z scores to perform the analyses on the whole sample. We explored potential sex differences in trauma exposure and did not find evidence of significant sex differences in history of sexual abuse or overall stress exposure levels.

#### recMDD cohort

The recMDD cohort consisted of 1,774 Caucasian individuals recruited at the MPIP in Munich, Germany and two satellite hospitals in the Munich metropolitan area (BKH Augsburg and Klinikum Ingolstadt): 756 controls and 879 cases diagnosed with recurrent major depression. Please see [61] for more details on sample recruitment and characterization and Table 1. A subset of *n* = 567 individuals was used in this manuscript.

All studies were approved by the local ethics committees and were conducted in accordance with the current version of the Declaration of Helsinki.

### Gene expression data

Whole blood RNA (Baseline and GR-response) from the MPIP cohort samples was collected using PAXgene Blood RNA Tubes (PreAnalytiX) and processed as described previously [62]. The RNA was then hybridized to Illumina HT-12 v3 and v4 expression Bead Chips (Illumina, San Diego, CA). Raw probe intensities were exported using Illumina’s GenomeStudio and further statistical processing was carried out using R version 3.2.1. All 29,075 probes present on both BeadChips (v3 vs. v4), excluding X and Y chromosomes as well as cross-hybridizing probes identified by using the Re-Annotator pipeline [62] were first filtered with a detection *p-*value of 0.05 in at least 50% of the samples, leaving 11,994 autosomal expression array probes. Subsequently, each probe was transformed and normalized through variance stabilization and normalization (VSN) [63]. Technical batch effects were identified by inspecting the association of the first principal components of the expression levels for all known batch effects and then adjusted using ComBat [63] with slide, amplification round, array version, and amplification plate column as fixed effects. The position of the gene expression probe and gene symbols were annotated using the Re-Annotator pipeline [62] based on GRCh37 (hg19) RefSeq [64]. Surrogate Variable Analysis (SVA) [65] was used to account for confounding as a result of batch effects, cell proportion or unknown factors using the SVA package in Bioconductor version 3.3. SVA was our preferred method over computational estimation of cell-type proportions as these methods are based on reference data sets that are not applicable to our data which was subject to the biological effects of dexamethasone, which impact cell-type proportions. However, we compared the significant SVs to the estimated fractions of different blood cell types derived from the residuals of the transcriptome-wide gene expression values using CellCODE [66], see Supplementary Fig. 1 for the SV correlations with blood cell count and known confounding factors. The log FC of gene expression was calculated as the difference in gene expression between post dexamethasone and baseline standardized to baseline. Gene expression data met the assumptions of all statistical models, and the variance of gene expression in males and females was estimated, similar and comparable (see https://github.com/jArloth/sex-specific-GR-response-Analyses).

### Genotype data and Imputation

Genotype data was generated for each cohort individually. Human DNA of the MPIP cohort samples was isolated from EDTA blood samples using the Gentra Puregene Blood Kit (Qiagen) with standardized protocols. Genome-wide SNP genotyping was performed using Illumina Human610-Quad (*n* = 173) and OmniExpress (*n* = 120) genotyping BeadChips according to the manufacturer’s standard protocols. recMDD cohort samples have been genotype on the Illumina-550 BeadChip and details on the genotyping methods have been previously published [61]. Quality control was conducted in PLINK 1.90b3s [67] or higher for each cohort and genotyping BeadChip separately. QC steps on samples included removal of individuals with a missing rate >2%, cryptic relatives (*PI-HAT* > 0.0125), an autosomal heterozygosity deviation (|*F*_het_ | >4 SD), and genetic outliers (distance in the ancestry components from the mean >4 SD). QC steps on variants included removal of variants with a call rate <98%, a MAF < 1%, and HWE test *p-*values ≤ 10^−6^. Furthermore, variants on non-autosomal chromosomes were excluded. Imputation was performed separately for each cohort and genotyping BeadChip with IMPUTE2, following phasing in SHAPEIT, using the 1000 genomes phase I reference panel (released in June 2014, all samples). QC of imputed probabilities was conducted in QCTOOL 1.4. Imputed SNPs were excluded if MAF < 1%, HWE test *p-*values ≤ 10^−6^, or an INFO metric <0.8. SNP coordinates are given according to hg19. SNPs were further processed in PLINK and variants were excluded if their MAF < 5%.

Genotyping of the LMU cohort was performed with the Infinium Global Screening Array BeadChip. Genotyping of the recMDD was performed with Illumina Human610-Quad BeadChips. Further detail on the genotyping and imputation methods used can be found in the individual papers LMU: [60] and recMDD: [61].

### Differential gene expression analysis (DEA)

To observe both dexamethasone-dependent changes in gene expression, and sex-stratified effects of dexamethasone, we ran the following models. First, we calculated the effect of sex on the difference in gene expression between baseline and post dexamethasone controlling for age, BMI, depression status, and cell type.

ΔGex ~ ß0 + ß1Sex + ß2age + ß3BMI + ß4depression + ß5cell type +ε

Second, a main effects linear model isolates the probes that are regulated by dexamethasone administration, controlling for sex, age, BMI, depression status, and cell type. Finally, the same main effect linear model was ran separately in males and females (not controlling for sex).

Gex ~ ß0 + ß1Dex + ß2Sex + ß3age + ß4BMI + ß5depression + ß6cell type +ε

### Expression quantitative trait loci analysis

The eQTL analysis was restricted to those SNPs within 1 Mb upstream or downstream.

For each gene expression array probe a linear model of the log fold change on gene expression was constructed between baseline and GR-response standardized to baseline and gender (only for the combined analysis). The residuals from the linear regression were used as phenotype values in the following analyses. PLINK v2 [67] was used to test for *cis-*association between all imputed SNPs and transcriptional response as previously described (Arloth et al., 2015).

ΔGex ~ ß0 + +ß1SNP + ß2Sex + ß3Age + ß4BMI + ß5depresson + ß6cell type +ε

We ran the same model, but separated for males and females for the sex-biased eQTL analysis.

ΔGex ~ ß0 + ß1SNP + ß2Age + ß3BMI + ß3depresson + ß4cell type +ε

Finally, for each set of sex-stratified etranscript gene expression array probe (identified by the models ran separately for males and females), the delta value between dexamethasone and baseline was predicted by the interaction of sex and eSNP, controlling for age, BMI, disease-state and SVAs (Supplementary Table 10).

ΔGex ~ ß0 + ß1Sex*SNP + ß2Age + ß3BMI + ß3depresson + ß4cell type +ε

As eQTL data were composed of two kinds of data: genotyping and expression data, we used two stages of multiple testing correction: (i) SNP level correction: for each *cis*-region (array probe) we performed a permutation test. The sample identifiers in the gene expression data were shuffled in order to preserve the structure in the genotype data (LD). A total of 500,000 permutations were carried out per probe and the empirical *P* values were adjusted using the Westfall-Young correction for the number of SNPs per probe, i.e., maxT procedure of Westfall-Young [68]. (ii) Probe level correction: *cis*-regions with an extensive LD structure will increase the number of false positive eQTLs [69]. Therefore, we applied the Benjamini-Hochberg method to correct the maxT adjusted *P* value significance by using only the most significant and independent SNPs per probe (tag SNPs). The number of tag eSNPs per *cis*-region was identified by LD pruning and “clumping“ the SNPs using the “clump” command in PLINK (using distance < 1 Mb and *r*^*2*^ ≤ 0.2 as setting). Each tag SNP forms a SNP bin, by aggregating all other SNPs into bins by tag SNP at *r*^*2*^ ≤ 0.2 and distance < 1 Mb, such that all SNPs within a given bin were correlated to their corresponding tag SNP, but not to any other tag SNP. We limited the false-positive SNP-probe pairs to less than 5% and therefore we considered the FDR analogue of the *P* value (*Q* value) < 5% as statistically significant.

### Power analysis

Given our different sample sizes of males and females, we determined our power for sex-stratified eQTL analyses. Given an effect size of the top eQTL for each analysis, we had 98% power in males, 57% power for females, and 79% for the combined sample with 0.07, 0.04, 0.02 as regression coefficients. For adequate power in the female only sample, we estimated that a sample of 382 would be required for equal power to the male analysis (98%) to detect *cis*-eQTLs. Power estimates were calculated using the G*power 3.19.4 application [70].

### Pathway analysis

The Bioconductor package TheGOstats was used to explore the gene ontologies of groups of transcripts over represented relative to all transcripts explored (*n* = 11,272 probes after quality control, or the gene ‘universe’). In terms of ontologies, we tested for biological processes and used the human genome wide annotation (org.Hs.eg.db). Due to high dependencies among GO terms, nominal *p-*values are reported. For descriptive purposes, the top gene ontologies were selected in the analysis of etranscripts overlapping with transcripts identified in BA25 in association with MDD.

### Genomic region annotation

eSNPs were overlapped with genomic annotation from UCSC for the hg19 genome build using *TxDb.Hsapiens.UCSC.hg19.knownGene* and *ChIPseeker* Bioconductor *R* packages.

#### Epigenetic enrichment analysis

To identify whether GR-response eSNPs were enriched for GR binding sites or co-localize with specific chromatin states, we used the Encode NR3C1 ChIP-seq data from GM12878 LCLs treated with dexamethasone (accession: GSE45638) and the 15-state ChromHMM [71] annotation of the Roadmap Epigenomics project among all cell lines of the blood and T-cell tissue group (*n* = 14 cell lines). We calculated the position-based overlap of the GR-response tag eSNPs and chromatin states for gender separately and compared the overlap observed with 1000 equal sized sets of baseline tag eSNPs adjusting for MAF. We used DeepSEA, a deep neural network pretrained with DNase-seq and ChIP-seq data from the ENCODE project, to predict the likelihood that GR-sex eSNPs exert regulatory effects on chromatin features comparing the reference to alternative SNP.

The coordinates of AR and ER binding sites were downloaded from Remap. There was no enrichment of sex-biased eSNPs for sex hormone receptors beyond baseline sex-biased eSNPs. To test for enrichment of TFs, we used the R package ReMapEnrich () using the 2018 Remap catalog on hg19.

We annotated the eSNPs using DeepSEA [72]. DeepSEA, a deep neural net- work pretrained with DNase-seq and ChIP-seq data from the ENCODE project, predicts the presence of histone marks, DNase hypersensitive regions (DHS) or TF binding for a given 1 kb sequence. The likelihood that a specific genetic variant influences regulatory chromatin features is estimated by comparing predicted probabilities of two sequences where the bases at the central position are the reference and alternative alleles of a given variant.

### DNA methylation data and meQTL analysis

For a subset of the reCMDD cohort (*n* = 567 individuals), genomic DNA was extracted from whole blood using the Gentra Puregene Blood Kit (QIAGEN). DNA quality and quantity of both was assessed with the NanoDrop 2000 Spectrophotometer (Thermo Scientific) and Quant-iT Picogreen (Invitrogen). Genomic DNA was bisulfite converted using the Zymo EZ-96 DNA Methylation Kit (Zymo Research) and DNA methylation levels were assessed for >480,000 CpG sites using the Illumina HumanMethylation450K BeadChips. Hybridization and processing were performed according to the manufacturer’s instructions. QC of methylation data, including intensity readouts, filtering (detection *p-*value > 0.01 in at least 75% of the samples), cellular composition estimated using *FlowSorted.Blood.450k* data and “estimateCellCounts” function, as well as beta calculation (“getBeta” function) were done using the *minfi* Bioconductor *R* package. CpG sites on sex chromosomes, CpG site probes found to have SNPs at the CpG site itself or in the single-base extension site with a MAF ≥ 1% in the 1000 genomes project EUR population and non-specific binding CpG site probes according to [73] were removed. We performed a re-alignment of the CpG site probe sequences using *Bismark*. This yielded 425,883 CpG sites for further analysis. The data were then normalized using functional normalization (“preprocessFunnorm” function in *minfi*) [74]. Technical batch effects were identified by inspecting the association of the first principal components of the methylation levels with plate and plate position. The data were then adjusted using “*ComBat”* function of the Bioconductor *R* package *sva*. CpG coordinates are given according to hg19.

For the meQTL analysis, linear regression models were fit for males and females separately and for each CpG site to test the relationship between the whole blood DNA methylation (beta values) and proximal SNP genotype (in dosage format) within 1 Mb up- or downstream of the SNP using the *R* package *MatrixEQTL* [75], in order to detect *cis*-meQTLs. Blood cell counts and age were included as covariates. Significance after multiple testing was adjusted using a false discovery rate (FDR) of 5%.

### Enrichment in Labonté et al. [29]

To test for enrichment of male and female GR-DE transcripts within male and female MDD transcriptional patterns in six brain regions, we used the ‘GeneOverlap’ R package to determine the significance of overlap from two lists based on the Jaccard index, given the size of common genes tested in the two data sets (*n* = 8683 genes). Enrichment for male and female GR eQTL associated etranscripts was tested in comparison to the overlap observed for baseline GR eQTL associated etranscripts based on odds ratios and *p*-values from Fisher’s exact test.

### GWAS enrichment analysis

The nominal GWAS results *p-*value < 0.05 of the Psychiatric Genomics Consortium (PGC) for different psychiatric disorders: schizophrenia (SCZ2) [76], bipolar disorder (BIP) [77], MDD (MDD1-3) [53, 54, 78], autism spectrum disorder (AUT) [79], attention-deficit/hyperactivity disorder (ADHD) [80], PTSD [81], Tourette syndrome (TS) [82] and cross disorder (CDG1&2) [83, 84] and non-psychiatric phenotypes: the Social Science Genetic Association Consortium (SSGAC) for educational attainment (EA) [85, 85], cannabis use [86], Type 2 diabetes (T2D) [87] and the Complex Trait Genetics Lab of the VU University of Amsterdam for intelligence [88] were used for comparison with our GR-response results. Thereby the overlap between the tag SNPs comprised in our eQTL bins and the SNPs identified by these studies were calculated. The enrichment eQTL-SNPs and GWAS risk-SNPs were tested in comparison with 1000 MAF-matched baseline tag eSNP sets.

### Transcriptional sensitivity profile score (TSPS)

TSPSs were based on the sets of significant GR-response tag eSNPs for males and females in the independent clinical LMU cohort. Of the 601 female GR-response eSNPs, 562 were available in the test cohort (with 57 proxy SNPs, *r*^2^ > 0.6), and of the 668 male, 650 (with 47 proxy SNPs, *r*^2^ > 0.6) used for calculation of the TSPS. Risk alleles were determined by the coefficient from the GR-response eQTL analysis, such that the alleles associated with higher absolute coefficients were coded as a risk allele. Absolute coefficients from the eQTL calculation were further included as weights. The scores were corrected for the number of SNPs. For eSNPs regulating multiple transcripts, we included each eQTL association and their beta coefficient. A higher TSPS thus denotes a larger number of alleles associated with larger GR-induced transcriptional response.

## Supplementary information

Supplementary Tables (all)

Supplementary Material

## Data Availability

Data from MPIP gene expression experiment are deposited at the GEO repository under GEO: GSE64930 and recMDD methylation under: GSE125105. Data analysis code is available at https://github.com/jArloth/sex-specific-GR-response-Analyses.
