# Supplementary material for: Sex differences in the genetic regulation of the blood transcriptome response to glucocorticoid receptor activation
Source: Transl Psychiatry. 2021 Dec 13;11:632. doi: 10.1038/s41398-021-01756-2 (PMC8669026; doi:10.1038/s41398-021-01756-2)
Supplement: Supplementary file 2 — Supplementary Material [file 41398_2021_1756_MOESM2_ESM.docx]

**Supplementary Material**

**Analysis of sex-biased GR-DE effects**

There may be transcripts which demonstrate a sex-biased effect of dexamethasone, which we tested by modeling sex as a predictor of the difference in gene expression between baseline and post dexamethasone administration standardized by baseline expression (see Methods), previously defined as GR-response values (Arloth et al., 2015). In this model, 26 transcripts demonstrated a significant effect of sex on dexamethasone change after multiple test correction (FDR< 0.05 **Supplementary Table 1**, and the majority (*n* = 15 transcripts; 57.69%) were already identified by the main effect model above or by the models stratified by sex. Across the 26 significant hits, the fold changes surpassed an absolute threshold of 0.2 for 7 transcripts, in which males and females demonstrated consistent directions of effects (i.e., one sex demonstrate a stronger effect than the other). The direction of the effect between baseline and dexamethasone was opposite for females compared to males for only two transcripts, but these fold changes did not surpass the fold change cut off for males or for females (see **Supplementary Table 1**).

Given our different sample sizes of males (*n* = 196) and females (*n* = 93), we tested the robustness of our results by using down-sampled sets of males matching female same size. We randomly drew 93 males from the full male sample to assess the number of significant transcripts found across 100 permutations. We found that the range of significant GR-DE transcripts found in males was between 3,125 and 3,251, with a mean of 3,178.2 $\pm$ 25, relative to 5,438 DE-GR transcripts identified with the full male sample and 6,568 DE-GR transcripts identified in females. All permutations yielded less transcripts than identified in the female sample of equal sample size.

**Effect size filtered transcripts and etranscripts**

**Supplementary Figure 2A** shows how these overlapping results across the general sample, males, and females with results are filtered by absolute log2 fold changes (> 0.2). A large proportion of the transcripts that were found by the main model to be significantly regulated by dexamethasone only surpassed the log2 fold change cut off in females (42.2%, *n* = 1705; shown in the shift to a larger number of transcripts unique to females in **Supplementary Figure 2A** relative to **Figure 1C**). In contrast, only 2 (0.12%) significant transcripts found in the main model surpassed the cut off uniquely in males. A minority of significant transcripts, 1,674 met the cut off across models. Taken together, these comparisons show that many of the transcriptional changes induced by dexamethasone identified in the main model were driven by larger effects in females. **Supplementary Figure 2B** similarly shows this trend, with a larger proportion of significant transcripts reaching the log2 fold change cut off from the female model relative to the general or male models.

We next assessed the size of the effects of those etranscripts that overlap with the transcripts from the differential expression analysis. Out of the 324 overlapping male etranscripts, 114 (35%) surpassed the absolute fold change threshold of 0.2 (range: -0.35 to 0.75), and 210 out of 333 (63%) of the overlapping female etranscripts surpassed the log2 fold change cutoff (range: -0.52 to 1.5). Thus, consistent with the GR-DE analysis, overlapping etranscripts in females exhibited larger fold changes relative to males (Wilcoxon p-value = 0.05).

Relative to the differential expression analysis, etranscripts meeting the fold change cut off were again largely found in males or females independently rather than the general, across sex analysis. Specifically, only six effect size filtered etranscripts were found in the general model and in males and females independently (**Supplementary Figure 3**). **Supplementary Figure 4** displays GR-DE effects identified in males (A) and females (B) independently.

**Baseline eQTLs**

When analyzing only baseline expression we found 4,202 general baseline eQTL bins (5,722 tag eSNPs of 167,885 correlated eSNPs and 2,909 gene expression probes and 2,550 genes, **Supplementary Table 8**). These results were compared to a larger publicly available database- the Biobank-Based Integrative Omics Study (BIOS) (>2,000 whole blood samples) and whole blood eQTLs from the Genotype-Tissue Expression project v8 (GTEx; 670 donors) (Consortium, 2020; Zhernakova et al., 2017). We found that 87% of our baseline eQTL genes overlapped with significant BIOS and 92% with significant GTEx eQTL genes.

We assessed male and female eQTLs separately at baseline and aimed to validate these baseline sex-biased eQTLs in existing data sets. We identified two times the number of eQTLs for males then for females, e.g. 1,960 eQTL bins (1,561 gene expression probes and 1,638 tag eSNPs; **Supplementary Table 9**) in females and 4,433 eQTL bins (2,561 gene expression probes and 3,209 tag eSNPs; **Supplementary Table 10**) in males only. Of note, for baseline eQTLs, the effects were significantly smaller for males relative to females (Wilcoxon p-value = 0.02). We found that 86% of our baseline male eQTL genes overlapped with nominal significant GTEx male eQTL genes (*p-value* < 0.05) and 84% of our baseline female eQTLs with nominal significant GTEx female eQTL genes. Thus, the sex differences we observed in eQTLs in the blood transcriptome at baseline are consistent with the extant literature, supporting the accuracy of identified sex differences.

**References**

Arloth, J., Bogdan, R., Weber, P., Frishman, G., Menke, A., Wagner, K. V., Balsevich, G., Schmidt, M. V., Karbalai, N., Czamara, D., Altmann, A., Trümbach, D., Wurst, W., Mehta, D., Uhr, M., Klengel, T., Erhardt, A., Carey, C. E., Conley, E. D., … Binder, E. B. (2015). Genetic Differences in the Immediate Transcriptome Response to Stress Predict Risk-Related Brain Function and Psychiatric Disorders. *Neuron*, *86*(5), 1189–1202. https://doi.org/10.1016/j.neuron.2015.05.034

Consortium, T. Gte. (2020). The GTEx Consortium atlas of genetic regulatory effects across human tissues. *Science*, *369*(6509), 1318–1330. https://doi.org/10.1126/science.aaz1776

Zhernakova, D. V., Deelen, P., Vermaat, M., Van Iterson, M., Van Galen, M., Arindrarto, W., Van’t Hof, P., Mei, H., Van Dijk, F., Westra, H. J., Bonder, M. J., Van Rooij, J., Verkerk, M., Jhamai, P. M., Moed, M., Kielbasa, S. M., Bot, J., Nooren, I., Pool, R., … Franke, L. (2017). Identification of context-dependent expression quantitative trait loci in whole blood. *Nature Genetics*, *49*(1), 139–145. https://doi.org/10.1038/ng.3737

Supplementary Figures

**Figure S1**: Correlation matrix of co-variants, surrogate variables (SVs) and estimated cell proportions based on CellCode.


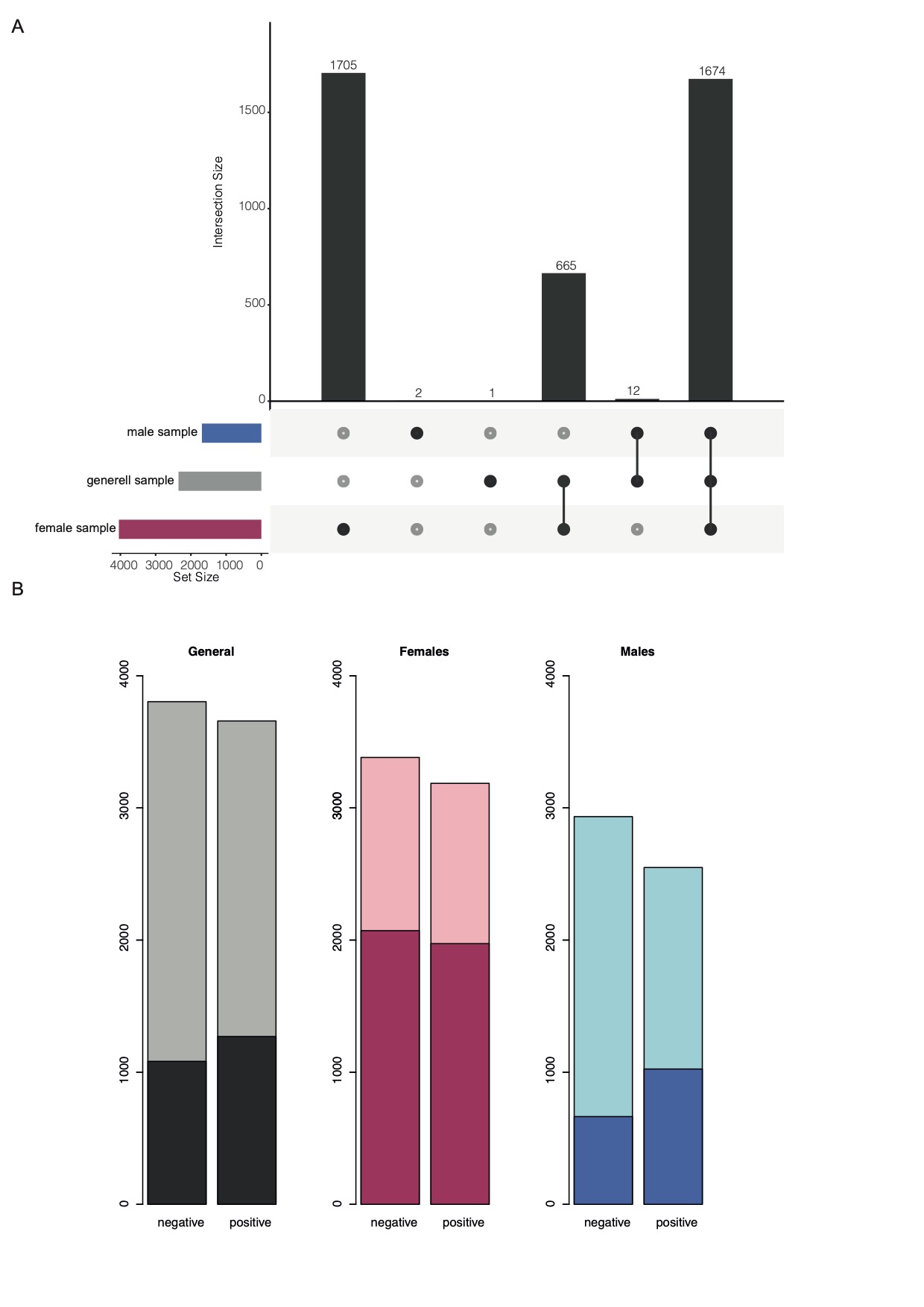


**Figure S2**: **A)** Upset plot of significant transcripts identified in combined sample, male sample, and female sample that meet absolute log_2_ FC threshold of > 0.2. **B)** Counts of significant negative and positive fold changes and significant changes surpassing an absolute log_2_ FC of 0.2 identified in the combined sample (significant gray, threshold overlaid in black), the female sample (significant pink, threshold overlaid in maroon) and the male sample (significant light blue, threshold overlaid in dark blue).


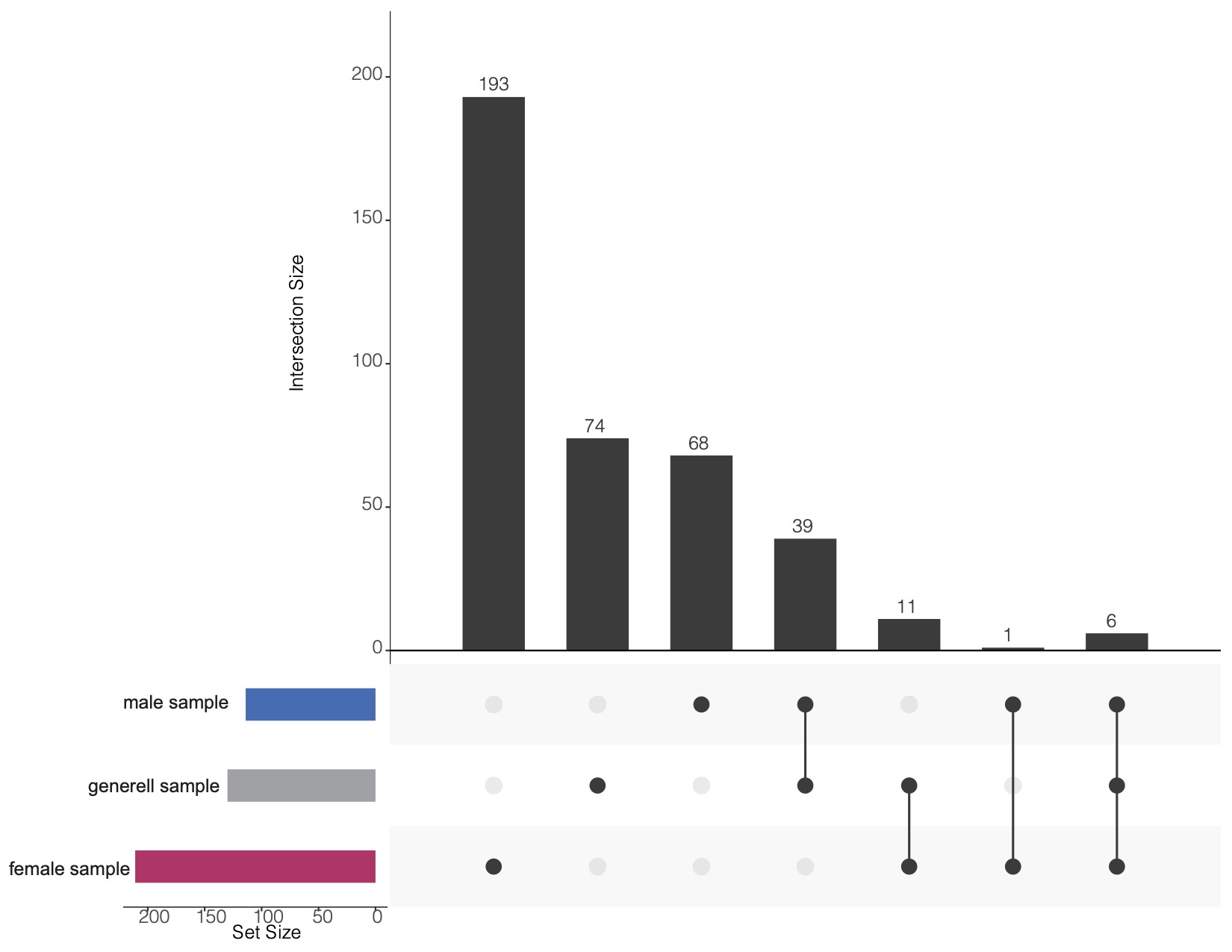


**Figure S3**: Upset plot of significant etranscripts identified in combined sample, male sample, and female sample that meet absolute log_2_ FC threshold of > 0.2.

**Figure S4**: Heat maps of log2 fold changes of the 5,483 GR-DE transcripts significantly differentially regulated in males (left) and 6,568 GR-DE transcripts in females (right).

**Figure S5**: Heatmap of enrichment results for GR-response tag eSNPs and predicted ChromHMM states for sex-stratified tag eSNPs. Colors displayed indicate fold enrichment and significant permutation *p-value* are written and were derived on the basis of 1,000 random sets of baseline eSNPs matched for allele frequency and size. We observed that male and female sex-stratified GR eSNPs were significantly enriched within repressed polycomb, bivalent enhancer and quiescent states (enrichment *p-value*s < 0.05) among the tissue group of blood and T-cells (*n* = 14 cell lines). For 70% of the blood tissue group cell lines, the state for ZNF genes and repeats (*n =*  2 cell lines), genic enhancers (*n =* 1 cell line) and active transcription start site (TssA and TssAFlnk, *n =* 3 cell lines) were significantly enriched only for female GR eSNPs, see Figure 3C. Male GR eSNPs were enriched for heterochromatin (*n* = 3 cell lines) and bivalent/poised TSS (*n* = 2 cell lines).

**Figure S6**: Relationships between eQTL effects and meQTL effects for **A)** females and **B)** males.

**Figure S7**: TSPS scores in correlation with individual depressive symptoms, as measured by the BDI and DIKJ.

Supplementary Table Legends

**Table S1**: Analysis of sex-dependent GR-DE effects. Note. Probe_Id = Illumina probe identifier; Estimate = regression beta value; Std. Error = standard error; Pr(>|t|) = nominal *p-value*; padj = FDR-adjusted *p-value*; Chr = chromosome; opp = whether or not the effect direction is opposite for males and females.

**Table S2**: Differentially regulated transcripts across model (GR-DE transcripts, full sample, males, and females). Note. Probe_Id = Illumina probe identifier; Estimate = regression beta value; Pval = nominal *p-value* full model; Padj = FDR-adjusted *p-value*s which represent the significance of a regression model; Chr = chromosome; log2FC = log_2_ fold change; FC = fold change.

**Table S3**: List of combined analysis GR-response cis-eQTL results. Note. Probe_Id = Illumina Identifier; SNP = rsID; CHR = chromosome; BP = base pair; A1= allele 1; A2 = allele 2; Location =genomic context location; nearByGene = gene in closest proximity; P_start = starting position of the probe; P_end = ending position of the probe.

**Table S4**: List of GR-response cis-eQTL results of females. Same column labels as S3.

**Table S5**: List of GR-response cis-eQTL results of males. Same column labels as S3.

**Table S6**: Results for interaction effect of SNP and sex on etranscript gene expression. Note. Probe_Id = Illumina Identifier; Estimate = regression beta value; Std. Error = standard error; Pr(>|t|) = nominal *p-value*; AdjP = FDR-adjusted *p-value*s which represent the significance of a regression model; SNP = rsIDl; Sex = male or female eSNP set.

**Table S7**: Results of pathway analysis of the significant etranscript sets of GR-response *cis-*eQTL identified in males and females. Note. GOBPID = the ID of biological process in GO database; Pvalue = nominal p value; ExpCount = expected number of genes in the enriched partition which map to this GO term; Count = number of genes in the enriched partition which map to this GO term; Size = number of genes within this GO Term; Term = Gene Ontology term description.

**Table S8**: List of baseline cis-eQTL results. Same column labels as S3.

**Table S9**: List of baseline cis-eQTL results of females. Same column labels as S3.

**Table S10**: List of baselines cis-eQTL results of males. Same column labels as S3.

modules.
